# The role of biopsies and autopsies in the diagnosis of cognitive impairment, with emphasis on small vessel diseases: A critical appraisal enriched by personal experience

**DOI:** 10.1590/1980-57642016dn11-040004

**Published:** 2017

**Authors:** Leila Chimelli

**Affiliations:** 1 Instituto Estadual do Cérebro Paulo Niemeyer – Laboratory of Neuropathology and Universidade Federal do Rio de Janeiro – Pathology Department, Rio de Janeiro, RJ – Brazil.; Universidade Federal do Rio de Janeiro, Pathology Department, Rio de Janeiro, RJ, Brazil, Universidade Federal do Rio de Janeiro

**Keywords:** cognitive impairment, cerebral small vessel diseases, vasculitis, cerebral and meningeal biopsy, autopsy, déficit cognitivo, doenças cerebrais de pequenos vasos, vasculite, biópsia cerebral e meníngea, autópsia

## Abstract

Acquired and hereditary microangiopathies cause cerebral small vessel diseases (CSVD) that impair cognition. The most frequent is primary angiitis of the CNS (PACNS), whose diagnosis remains challenging, requiring a multidisciplinary approach. Secondary vasculitis, CADASIL, miscellaneous microangiopathies and lymphomas, also cause cognitive impairment. Despite the fact that the need for biopsy has decreased in the era of new neuroimaging methods, biopsies that include small leptomeningeal and parenchymal arterial vessels still remain the gold standard to diagnose PACNS and other CSVD, and to exclude mimics such as infections and malignancies. New approaches for pathological consequences relevant to vascular cognitive impairment such as silent brain lesions, microinfarcts, microbleeds and subtle loss of microstructural integrity, may be detected in autopsies. This article addresses the role of biopsies and autopsies for the diagnosis of cognitive impairment related to small vessel diseases or other inflammatory/ischemic processes, and presents a critical appraisal based on personal experience.

## RESUMO.

As microangiopatias adquiridas e hereditárias causam doenças cerebrais de vasos pequenos, que comprometem a cognição. A mais frequente é a vasculite primária do sistema nervoso central, cujo diagnóstico continua desafiador, exigindo abordagem multidisciplinar. Vasculite secundária, CADASIL, microangiopatias diversas e linfomas também causam deficiência cognitiva. Apesar da necessidade de biópsia ter diminuído na era de novos métodos de neuroimagem, as biópsias que incluem pequenos vasos arteriais leptomeníngeos e parenquimatosos continuam sendo o padrão-ouro para o diagnóstico de vasculites primárias, afastando situações que as mimetizam, como infecções e neoplasias. Novas abordagens sobre as alterações teciduais de origem vascular relevantes para comprometimento cognitivo, como lesões cerebrais silenciosas, microinfartos, microssangramentos e perda leve de integridade micro estrutural, podem ser detectadas em autópsias. Este artigo aborda o papel das biópsias e autópsias para o diagnóstico do comprometimento cognitivo associado a doenças cerebrais de pequenos vasos ou outros processos inflamatórios/isquêmicos, e apresenta uma avaliação crítica baseada em experiência pessoal.

## INTRODUCTION

In its broadest sense, “microcirculation” includes parenchymal and leptomeningeal arterioles, venules, and capillaries. Arbitrarily, CNS small vessels are defined as small veins and perforating arteries derived from the circle of Willis with diameters ranging from 40 to 400 µm.^1^

“Cerebral Small Vessel Diseases (CSVD)” or microangiopathies involve intraparenchymal vessels, including predominantly arterioles, mostly end arteries with limited collateral anastomoses up to the capillary network. Exceptions to this rule are some vasculites involving larger vessels, which are conventionally included within this group of conditions. CSVD are associated with a variety of ischemic and/or hemorrhagic manifestations. Cerebral microangiopathies such as arteriolosclerosis, lipohyalinosis and cerebral amyloid angiopathy (CAA), commonly produce intraparenchymal hemorrhage, but arteriosclerosis and lipohyalinosis are also associated with cerebral infarcts, especially small lacunar infarcts; CAA is also occasionally associated with ischemic lesions.^2^^,^ 3

The development of new neuroimaging methods has enhanced awareness of these important causes of neurologic morbidity. Correlation between neuroimaging data and neuropathology is important and may help identify characteristic patterns of abnormality such as small hemorrhages, microbleeds, lacunes, status cribrosus and secondary changes in white matter (leukoencephalopathies).

### CSVD associated with cognitive impairment/dementia.

Ischemic lesions within subcortical white matter, as well as secondary leukoencephalopathies resulting from necrosis of adjacent tissue, may contribute to cognitive impairment and dementia. Though dementia may result from widespread arteriosclerosis or CAA in the brain, and be associated with varying degrees of cerebral ischemia, two distinct entities should be considered in this context: Binswanger's subcortical arteriosclerotic leukoencephalopathy (BSL) and Cerebral Autosomal Dominant Arteriopathy with Subcortical Infarcts and Leukoencephalopathy (CADASIL).^1^^,^ 3 ^-^ 5

**BSL** is a disease of elderly hypertensive patients, characterized by a slowly progressive condition, typically showing stepwise deterioration and episodes of partial recovery. Disorders of memory, mood, and cognition, as well as occasional focal motor signs and less commonly, a pseudobulbar syndrome in association with deterioration of gait and loss of sphincter control, can be observed. The brain may show asymmetric poorly delineated regions of leukomalacia, lacunar infarcts in the centrum semiovale, with preservation of the overlying cerebral cortex. Histology of white matter shows thickened arteries with an ‘onion skin' appearance and scattered chronic inflammation with macrophage/microglial infiltrate around blood vessels. Transition between relatively preserved white matter (containing sclerotic arteries with severe adventitial fibrosis), diffuse myelin pallor, sometimes poorly defined white matter cavitation, and gliosis may be seen. Meningeal arteries are sometimes surrounded by hemosiderin, suggesting previous hemorrhage.^3^

**CADASIL** resembles BSL, but is distinguished by its familial nature and the deposition of abnormal material onto vessel walls. Individuals with CADASIL have missense mutations of the Notch 3 gene on chromosome 19q12. The onset is usually in the fifth or sixth decade of life and the presentation includes migraine, repeated subcortical ischemic strokes and dementia. Affected vessels predominate in the leptomeninges and deep white matter, but are also seen in basal ganglia, thalamus, pons and the spinal cord. There is fibrous vascular thickening of the arteriolar wall with stenosis of the lumen, degeneration of smooth muscle cells, and replacement of the media by eosinophilic, weakly PAS-positive, Congo red-negative granular material. Specific changes include granular basophilic deposits in the media of arterioles (“Granular Osmiophilic Material, GOM” on electron microscopy) and accumulation of the extracellular domain of Notch3 on immunohistochemistry. Secondary brain changes include lacunar infarcts, leukoencephalopathy, hemorrhages (uncommon) and status cribrosus, with apoptosis of endothelial cells. Involvement of the cerebral cortex is uncommon, although cortical atrophy may be associated with neuronal apoptosis and contribute to cognitive decline, despite the fact that CADASIL is considered a typical example of subcortical dementia. The GOM on the vessel walls can also be detected in skin biopsies, adjacent to smooth muscle cells in the walls of dermal arterioles.^1^^,^ 3 ^,^ 6 ^,^ 7

Other non-amyloid hereditary CSVD include Retinal Vasculopathy with Cerebral Leukodystrophy (RVCL), small vessel disease mapping to chromosome 20q13, autosomal recessive CARASIL, Leukoencephalopathy Calcifications and Cysts (LCC), Hereditary Endotheliopathy with Retinopathy, Neuropathy and Strokes (HERNS), Cerebroretinian Vasculopathy (CRV), Mitochondrial Encephalopathy Lactic Acidosis and Stroke (MELAS), and others.^7^^,^ 8

### Central Nervous System (CNS) vasculitis.

Angiocentric inflammation of the wall of cerebral blood vessels and/or the perivascular space are increasingly recognized and are important causes of CSVD.^9^^,^ 10 Cerebral vasculitis is a disease that may involve the CNS exclusively (primary angiitis of the CNS) or part of a systemic disorder (secondary vasculitis, see below).

**Primary angiitis of the CNS (PACNS)** can occur at any age, including childhood, but tends to occur in the ‘middle-aged' and elderly, with peak usually in the 5^th^/6^th^ decades of life. Males are more commonly affected than females. It is probably multifactorial in origin, an unusual response to autoantigens or viral infection, and varicella-zoster virus has been implicated in some cases. A range of neurologic symptoms include headaches (the most common symptom), diffuse neurologic dysfunction, and ischemic or hemorrhagic stroke; it should be considered in patients with cerebral ischemia affecting different vascular territories in association with inflammatory changes in the CNS. A typical presentation is insidious onset of headaches with cognitive impairment but the initial presentation can be polymorphic and non-specific. The diagnosis of PACNS remains challenging, relies on the presence of isolated neurological symptoms along with evidence of brain vessel involvement and the exclusion of all mimicking conditions or secondary causes of CNS vasculitis. Therefore, a multidisciplinary approach is required to investigate a broad differential diagnosis. Magnetic resonance imaging (MRI) has high sensitivity but poor specificity; magnetic resonance angiography (MRA) and angio-CT are noninvasive, but their resolution is often inadequate for detecting the vasculitic changes in PACNS. The typical angiographic finding consists of alternating areas of stenosis and dilation, referred to as beading. However, since it affects mainly small cerebral arteries, the changes may not be seen properly even with the resolution of conventional angiography. Because the specificity can be as low as 30%, some patients with normal angiography were later found to have PACNS on brain biopsy, which remains the gold standard procedure to highlight the vasculitis process but is performed in less than half of patients in practice and can reveal some negative results (see below). In patients with non-contributive biopsy or without biopsy, diagnosis relies on demonstration of vascular involvement on neurovascular imaging, such as digital subtraction angiography (DSA), MRA or cerebral angio-CT scan.^10^^-^ 13 ^,^ 15

Secondary CNS vasculitis due to systemic autoimmune diseases, including those which involve small and medium-sized vessels (polyarteritis nodosa), systemic lupus erythematosus, Wegener's granulomatosis with polyangiitis, Behçet's syndrome, Sjögren's syndrome, sarcoidosis, vasculitis associated with anti-neutrophil cytoplasm antibodies (ANCA), rheumatoid arthritis, and infectious vasculitis, should be considered in the setting of extracranial symptoms. HIV-1, CMV or VZV, Syphilis, Borrelia *burgdorferi*, Mycobacterium *tuberculosis*, and various other bacteria and fungi are among systemic infections that may present with diffuse and focal neurologic deficits.^12^^-^ 15

Since encephalopathy is a frequent feature of PACNS, workup of these patients should include evaluation for paraneoplastic conditions and antibody-mediated inflammatory brain diseases including NMDAR encephalitis and voltage-gated potassium channel antibodies with limbic encephalitis. Abnormal angiographic findings may help distinguish PACNS from these conditions.^1^^,^ 3 ^,^ 16

Miscellaneous microangiopathies include thrombotic thrombocytopenic purpura, siderocalcinosis and ferruginization of microvessels, cortical superficial siderosis, Sneddon syndrome (cerebral infarcts + livedo reticularis) and others. Their clinical manifestations are variable, ranging from none (in vascular siderocalcinosis) to widespread micro-infarcts. Reversible Cerebral Vasoconstriction Syndrome should also be included in the differential diagnosis of microangiopathies.^15^

Angiocentric inflammatory/neoplastic infiltrations (Lymphomatoid granulomatosis – LG; and Lymphomas) can also be included in this group. LG is a controversial entity that appears to link vasculitis with primary CNS (angiotrophic) lymphoma and predominantly affects lung, but involves the CNS in 10-15% of patients. Pathologic features include a polymorphous angiocentric, angiodestructive cellular infiltrate, in which a polymorphous transmural infiltrate of atypical leukocytes is often combined with fibrinoid necrosis of parenchymal arteries and arterioles, thrombosis and with surrounding brain infarction. CNS lymphomas, including primary intravascular lymphoma, can mimic the radiographic findings of PACNS. A brain biopsy can help to identify these disorders.^3^

## USEFULNESS OF BRAIN BIOPSIES TO DIAGNOSE COGNITIVE IMPAIRMENT RELATED TO CSVD

Brain biopsies may be required in patients with cognitive impairment included in the category of cryptogenic neurological disease, which comprises acute or chronic neurological deterioration of unknown etiology (e.g. chronic meningitis), CNS vasculitis, and atypical dementia. This is a particularly challenging subgroup of diseases in which noninvasive diagnostic options are frequently unrevealing. Therefore, it is often necessary to obtain histological evidence before initiating potentially toxic therapies. Although brain biopsy is considered an invasive diagnostic modality, it carries a very low risk (1% or less) of neurologic deficit and is often essential in obtaining the correct diagnosis and avoiding inappropriate treatment. It can help not only to assist in the identification of angiitis, when clinically suspected, but also to exclude mimics such as infections and malignancies.^11^^,^ 15

In the early days of PACNS, brain histopathology findings were entirely based on autopsy results. Over the years, more centers worldwide have started to perform elective brain biopsies in children and adults with suspected PACNS and negative angiographic findings, with a diagnostic yield of 70-75%. The leptomeningeal vessels, mainly arterioles, are more commonly involved than the parenchymal vessels and when the leptomeninges are sampled the diagnostic sensitivity rises to 87%. Therefore, it is recommended that when vasculitis is suspected and biopsy is to be performed, it should target an area of imaging abnormality, and leptomeninges and parenchyma should be included to maximize the diagnostic yield. In absence of a clear target lesion based on imaging, a ‘‘blind'' cortical and leptomeningeal biopsy from the frontal lobe or temporal lobe tip of the non-dominant hemisphere is suggested, despite the risk of negative diagnostic results. One should be aware that biopsy is useful not only to provide the diagnosis of vasculitis, but also to exclude alternative diagnoses, such as infectious encephalitis, primary CNS lymphoma, brain abscess, demyelination, and a variety of other tumor and non-tumor conditions.^10^^,^ 11 ^,^ 16

In a meta-analysis evaluating the role of brain biopsies in patients with cryptogenic neurologic disease, involving several centers, the highest diagnostic yield was in patients with PACNS. The diagnostic success rate was much higher in the latter period (74%) in comparison with the earlier period (43%), therefore an improvement in the diagnostic yield of biopsies has been observed in recent years. According to the authors, this may be explained by the improved surgical techniques or the fact that fewer, but more selective patients were sent for brain biopsy because of improved diagnostic techniques. The presence of a radiological target was associated with the higher diagnostic yield. The value of diagnostic biopsies in terms of clinical impact is debatable if the identified disease is not treatable. However, according to the authors, a nondiagnostic biopsy can still be of clinical relevance. The exclusion of certain conditions may permit withdrawal of potentially ineffective treatments and/or give clinicians the confidence to proceed with treatments that are otherwise contraindicated. Even patients with a definite diagnosis, but no impact on clinical management, may gain emotional comfort simply with information from the biopsy. Clinicians should take individual clinical scenarios into consideration when discussing the risks and benefits of brain biopsy with their patients.^16^

The quality of the elective brain biopsy material submitted to the pathologist is of great importance: it should contain leptomeninges, gray and white matter. If only leptomeninges are biopsied the yield accuracy in finding the lesion decreases by 20%. Immunosuppressive treatment prior to and at the time of brain biopsy confounds the histologic findings. In addition, brain biopsy may be falsely negative when unaffected tissue is sampled; although the risk is small, it is not a procedure that can easily be repeated over time. Small intraparenchymal hematomas at the biopsy site may occur (4.9%) and also permanent neurological sequelae, albeit at a lower frequency (less than 1%).^11^^,^ 17

Regarding the interpretation of brain and meningeal biopsies, although the histopathology of PACNS is quite variable, the three patterns, granulomatous, diffuse lymphocytic and necrotizing, may be seen (see below). Since lymphocytic infiltrates within vessel walls can be found in the context of many different CNS pathologies and represent a nonspecific finding, the presence of a sparse perivascular mononuclear cell infiltrate is not sufficient for the diagnosis of vasculitis. Marked segmental transmural lymphocytic inflammation (and occasional plasma cells), sometimes destroying the wall elements, in the absence of significant parenchymal inflammation, is required. Focal granulomatous inflammation and fibrinoid necrosis may occasionally be present. Granulomatous vasculitis is characterized by vasculocentric destructive mononuclear inflammation associated with well-formed granulomas (through the vascular wall from the intima to the adventitia) and/or multinucleated giant cells. b-A4 amyloid deposition is mostly seen in the granulomatous vasculitis group. Acute necrotizing vasculitis, the least frequent pattern, involves predominantly small muscular arteries and is associated with disruption of the internal elastic lamina, with transmural fibrinoid necrosis and acute inflammation. Acute neutrophilic inflammation per se is also insufficient for the diagnosis of PACNS. Necrosis of the vascular walls should be considered in vessels distal from areas of acute tissue necrosis/hemorrhage, where vascular wall necrosis and neutrophilic infiltration are often seen as a secondary phenomenon. Amyloid depositions can be seen in adult PACNS, but are absent in childhood PACNS.^11^^,^ 17 It is worth mentioning that Amyloid b-related angiitis, caused by amyloid-b peptide deposition in the brain leading to vessel wall breakdown, is a subset of PACNS but patients tend to present the condition at an older age.^18^ The histological differential diagnosis of PACNS includes sarcoidosis and infectious meningitides, but these have distinctive patterns such as viral inclusions, neuronophagia, parenchymal inflammation, and/or demyelination and are not restricted to blood vessels. The angiocentric accumulation of lymphoid cells may also bear a superficial resemblance to primary CNS lymphoma, especially well-differentiated lymphoplasmacytoid variants, but infiltrates of neoplastic cells tend to be more extensive, may be partly necrotic, contain much more reticulin, lack a granulomatous component, and have a characteristic immunophenotype.^11^

In the diagnosis of PACNS it is important to take into account that discrepancies between biopsy and angiography may occur. Brain biopsy may have the highest diagnostic yield (74.7%) when the indication is to evaluate for suspected PACNS, but may have false negative results and PACNS be later diagnosed based on a combination of clinical course and radiological evidence. In false-negative brain biopsies, lesions can have irregular involvement or be inaccessible. Diagnostic information from angiograms and CNS biopsies are, therefore, often complementary, and the diagnostic likelihood of a brain biopsy can be increased by targeting the abnormality with imaging and combining parenchymal and leptomeningeal sampling. It has also been shown that CNS lymphoma and intravascular lymphoma can be difficult to distinguish from PACNS based on clinical presentation and imaging characteristics alone. Therefore, it is recommended that, in patients with suspected PACNS, accurate diagnosis should not rely on any single study, but instead on careful correlations among clinical, radiographical, and pathological findings.^10^

For Chronic Meningitis of Unknown Etiology, which can be caused by a variety of infectious and noninfectious processes and is arbitrarily defined as meningitis that lasts for at least 4 weeks without any improvement, the brain biopsy may have a low diagnostic yield. Improved serological and CSF testing for herpes simplex virus and other viruses precludes the need for brain biopsy. Similarly, autoimmune and paraneoplastic causes of encephalitis, such as anti-NMDAR, anti-LGI1/VGKC, and anti-Hu, have well-validated diagnostic antibody tests in either serum or CSF. Brain biopsy is therefore not recommended unless thorough antibody testing and cancer screening are unrevealing. A definite diagnosis can be achieved by sampling the meninges alone, particularly when meningeal enhancement is demonstrated on MRI.^16^

In patients with Atypical Dementia, the utility of tissue diagnosis remains controversial. In most cases, the etiology of dementia can be determined accurately by clinical criteria alone, and brain biopsy is unnecessary. It may be used to rule out treatable causes of dementia such as encephalitis or chronic meningitis, but one of the major criticisms of brain biopsy in patients with dementia is that the diagnoses established are frequently of diseases without current treatment options, such as Alzheimer's disease (AD) and Creutzfeldt Jacob disease (CJD). Consequently, biopsy is not recommended in this subset of patients at this time. If the clinical presentation is consistent with CJD but the biopsy is nondiagnostic, the probability of the patient having CJD is again lowered, provided that the biopsy has been performed in the cerebral cortex (comments below).^8^^,^ 16

Concluding, brain biopsy remains a useful tool in establishing a definite diagnosis in patients with cryptogenic neurological disease. Despite other diagnostic advances and even though sensitivity of biopsy may be suboptimal, strategies can be adopted to increase the likelihood of obtaining a diagnostic biopsy, including targeting sites of imaging abnormality and combining parenchymal and leptomeningeal sampling. The indication to evaluate for suspected PACNS is associated with the highest diagnostic yield.^16^^-^ 19

### New approaches for pathological consequences relevant to vascular cognitive impairment (VCI) and vascular dementia (VaD) detected in post-mortem tissues.

In addition to the obvious focal destructive effects of infarcts and foci of hemorrhage, there is increasing appreciation of the more widespread or remote damage to neurons, synapses and white matter tracts in VCI and VaD. Cortical disconnection as a result of diffuse damage to cerebral white matter that affects the integrity of cortical association fibers and subcortical tracts seems to be a particularly important contributor to cognitive decline.^20^

This is likely to be relevant to cognitive impairment in neurodegenerative, as well as vascular disease, partly because neuronal degeneration leads to axonal loss, but also because cerebral hypoperfusion is an intrinsic component of some neurodegenerative diseases involving cortical neurons. Diffuse white matter damage is well demonstrated by diffusion tensor MRI but difficult to assess and quantify histologically. However, there is some pathological evidence of the important contribution of ischaemic white matter damage to cognitive impairment. VCI is a broad concept that covers the full spectrum from vascular mild cognitive impairment (vascular MCI) to VaD and includes cases with mixed pathologies, such as mixed vascular and AD-type pathologies.^8^^,^ 21

**Silent Brain Lesions (SBL)** of vascular origin are common in elderly people, their prevalence increasing from ≈10% to 40% in subjects aged 65 and 90 years, respectively, and whose prevalence is even higher in patients with vascular risk factors. Most SBL are lacunes attributable to SVD. Thalamic infarcts are associated with a decline in memory performance, whereas non-thalamic infarcts are associated with a decline in psychomotor speed.^20^^-^ 23

### Microinfarcts.

Only recently, investigators have recognized the impact of cerebral microinfarcts (CMI) on cognitive function and risk of dementia. CMI are small ischemic lesions not visible to the naked eye (<1 mm) but detected microscopically during pathological examination, where they may be cystic or incomplete. These lesions represent the most widespread form of brain infarction and are generally attributed to SVD, although other mechanisms, such as microemboli, cerebral hypoperfusion or vasoconstriction, are also discussed as potential causes. The presence of 1 or 2 CMI in routine neuropathological specimens implies the presence of hundreds throughout the brain. CMI may be located in cortical or subcortical regions and are particularly common in patients with VCI. However, they are also frequent in AD patients and in unselected elderly people. Quantifying CMI *in vivo* remains a challenge: they are best detected on ultrahigh-field MRI at 7 T, but may occasionally be seen on conventional 3T scans. MRI is much more sensitive for detecting acute small infarcts detected on diffusion-weighted imaging.^20^^,^ 22 ^,^ 24

**Microbleeds (MBs)** are small, round, well-defined foci of MRI signal void appearing black on gradient echo T2-weighted scans. They are detected in 10% to 15% of elderly subjects, in ≤80% of patients with VaD, are generally considered a manifestation of SVD and have been related to focal deposits of iron-positive blood breakdown products. The mechanisms by which MBs affect cognition are still debated, as is the impact of MB location on cognitive impairment or dementia. There is some evidence that MBs disrupt structural connectivity and, hence, network function, although the underlying pathology and mechanisms may be more heterogeneous.^20^^,^ 25

**Subtle Loss of Microstructural Integrity** is among the earliest manifestations of SVD. These early stages are not detected by conventional MRI but are captured by diffusion tensor imaging, the best technique to correlate it with cognitive function and decline. Moreover, the same measures enable identifying individuals at risk for developing cognitive decline even when measured within tissue appearing normal on conventional MRI. This may allow for a completely new treatment perspective because current approaches usually fail when treatment is started in patients with advanced pathology. A valuable addition in this context has been the introduction of novel tools to quantify microstructural tissue damage across major white matter tracts in an automized way.^20^

### Personal experience with meningeal or cerebral biopsies to diagnose cognitive impairment related to small vessel diseases or other inflammatory/ischemic processes.

After examining specimens from neurosurgical procedures for many years, it was possible to observe that in about 5% of cases, the meninges or superficial brain tissue were biopsied with the clinical hypotheses of inflammation, vasculitis or an ischemic process. Of these, vasculitis (mostly PACNS) was confirmed in about 35% of the cases. Other findings were nonspecific inflammatory infiltrates, particularly lympho-histiocytic, sometimes associated with gliosis, necrosis, ischemic neurons and neovascularization, indicating an organizing process. Granulomatous meningitis, with or without an infectious agent, was occasionally seen, confirming tuberculosis or suggesting sarcoidosis.

A B-cell lymphoma was confirmed in some of these specimens, while occasional infectious diseases, associated or otherwise with immunodeficiency (toxoplasmosis, tuberculous meningitis, and less frequently progressive multifocal leukoencephalopathy, which requires a white matter sample), were also diagnosed. Other diseases related to small vessel involvement included CADASIL and secondary microvasculitis possibly related to auto-immunity/paraneoplastic diseases. Normal tissue was occasionally biopsied.

These biopsies are usually required by neurologists and glass slides and paraffin blocks are often received for second opinion, after inconclusive reports derived from non-specialist surgical pathologists. Serial sections are frequently necessary to look for the lesion and on a few occasions disclose the pathological process, usually a lymphocytic vasculitis. Alternatively, these reviewed slides disclose only gliosis, edema, scattered lymphocytes and macrophages, which is usually frustrating for the pathologist who reports the results, for the clinician and surgeon who receive the reports, but mainly for the patients. However, as mentioned above, some comfort may be obtained, after possibly excluding more serious lesions.

Biopsy of the dura mater usually does not contribute to the diagnosis. On one occasion, in which rare granulomas were seen after serial sections and no infectious agent was identified, the possibility of sarcoidosis was suggested. The dura mater was otherwise always normal.

Although biopsies are usually not performed to diagnose CJD, the ideal approach is to examine the whole brain obtained from autopsies, in rare situations a biopsy is performed. Unfortunately, in many cases it does not help the diagnosis, because of the limitation in amount of tissue. Spongiform changes can sometimes be patchy and may not be represented in the biopsied specimen. In addition, it is very important to inform the surgeon that the biopsy should include the cerebral cortex. Once the biopsy was performed in the white matter; therefore it was not contributive to the diagnosis, since the disease is basically cortical. Immunostaining with antibody for PrP is mandatory to confirm the diagnosis, because spongiosis is a rather nonspecific change, occurring in other neurodegenerative or metabolic diseases, and may even be due to fixation artifact.

When the biopsy is performed with the clinical hypothesis of CADASIL, it is necessary to fix part of the tissue in glutaraldehyde for electron microscopy, unless the antibody for Notch3 is available to perform immuno-histochemistry, when formalin-fixed tissue is sufficient. When these methods are not available, one can describe the presence of thickened arteriolar walls, sometimes with granular PAS positive material, which may suggest, but not confirm the disease, since other microangiopathies can present with thickened vessels. It is important to remind the surgeon that the main lesion is in the white matter. However, the neurologist should be aware that skin biopsy, a much less invasive procedure, is also useful to diagnose CADASIL.

CAA is easily confirmed when thickened hyaline vessels walls are included in the biopsy. In most cases it is associated with fresh hemorrhage and Congo red staining is sufficient for the diagnosis.

It is not uncommon to find only reactive gliosis and occasional small vessels with thickened walls, sometimes surrounded by few lymphocytes and macrophages, and even hemosiderin pigment, indicating previous hemorrhages. These findings are totally non-specific, but within the whole context, when all clinical and neuroimaging information is available, it is possible to exclude some diagnoses and suggest others. The observation of ischemic (red) cortical neurons is indicative of an ischemic process and some diagnostic possibilities can be postulated.

One last comment about tissue sampling that is not representative of the lesion. Although intraoperative assessment of tissue representativeness has been recommended to increase diagnostic yield, it may consume precious tissue (particularly when the sample is small) that will not then be available for paraffin embedding, additional sections, special staining and immunostaining, all important to increase diagnostic yield. Therefore, each individual sample should be evaluated to decide whether it is worthwhile or not to prepare a smear or frozen section to evaluate tissue representativeness.

In conclusion, the pathologists can help to diagnose the cause of cognitive impairment, provided that the sample is representative of the lesion, and a close clinico-pathological and neuroimaging correlation has been established.
